# Central and peripheral venous lines-associated blood stream infections in the critically ill surgical patients

**DOI:** 10.1186/1750-1164-6-8

**Published:** 2012-09-04

**Authors:** Mohamed Ali Ugas, Hyongyu Cho, Gregory M Trilling, Zainab Tahir, Humaera Farrukh Raja, Sami Ramadan, Waseem Jerjes, Peter V Giannoudis

**Affiliations:** 1 Barts and the London School of Medicine and Dentistry, Queen Mary, University of London, London, UK; 2 University College London Medical School, London, UK; 3 Department of Surgery, Al-Yarmouk University College, Baghdad, Iraq; 4 School of Medicine, University of Leeds, Leeds, UK

## Abstract

Critically ill surgical patients are always at increased risk of actual or potentially life-threatening health complications. Central/peripheral venous lines form a key part of their care. We review the current evidence on incidence of central and peripheral venous catheter-related bloodstream infections in critically ill surgical patients, and outline pathways for prevention and intervention. An extensive systematic electronic search was carried out on the relevant databases. Articles were considered suitable for inclusion if they investigated catheter colonisation and catheter-related bloodstream infection. Two independent reviewers engaged in selecting the appropriate articles in line with our protocol retrieved 8 articles published from 1999 to 2011. Outcomes on CVC colonisation and infections were investigated in six studies; four of which were prospective cohort studies, one prospective longitudinal study and one retrospective cohort study. Outcomes relating only to PICCs were reported in one prospective randomised trial. We identified only one study that compared CVC- and PICC-related complications in surgical intensive care units. Although our search protocol may not have yielded an exhaustive list we have identified a key deficiency in the literature, namely a paucity of studies investigating the incidence of CVC- and PICC-related bloodstream infection in exclusively critically ill surgical populations. In summary, the diverse definitions for the diagnosis of central and peripheral venous catheter-related bloodstream infections along with the vastly different sample size and extremely small PICC population size has, predictably, yielded inconsistent findings. Our current understanding is still limited; the studies we have identified do point us towards some tentative understanding that the CVC/PICC performance remains inconclusive.

## Introduction

Critically ill surgical patients are at an increased risk of actual or potentially life-threatening health complications. Common complications include ventilation acquired pneumonia (VAP), gastrointestinal bleeding, deep vein thrombosis, hyperglycaemia, arrhythmias, acute renal failure and venous catheter-related bloodstream infection (CRBSI) [1]. Nonetheless, central venous catheters (CVC) and peripherally inserted central catheters (PICC) form a key part of care in any critically ill patient by providing central venous access; common indications include measurement of central venous pressure and administration of medications, nutrients and fluids including blood products [2].

CVCs are introduced through the internal jugular, subclavian, axillary or femoral vein whilst PICCs are inserted into either the cephalic, basilic, or brachial vein of the arm. PICCs are a much less-invasive alternative to traditional CVCs, provide prolonged intravenous access and are associated with fewer traumatic complications.

Poor-technique CVC insertion can cause pneumothorax, whilst both central and peripheral lines can develop catheter occlusion, thrombosis, phlebitis, endocarditis, metastatic infections (i.e. brain or lung abscesses, endophthalmitis and osteomyelitis) and CRBSI [2,3].

CRBSI is an important cause of morbidity and mortality in the surgical acute care unit (High Dependency or Intensive Care Units) and accounts for 10-20% of hospital-acquired infections in the UK [4]. Organisms typically originate from the skin flora and include *coagulase-negative staphylococci, staph aureus,* aerobic *gram-negative bacilli* and *candida albicans*[5,6].

This systematic review aims to summarise the current evidence on incidence of CRBSI in central and peripheral lines in critically ill surgical patients, and outline pathways for prevention and intervention.

## Materials and methods

**Figure 1:**
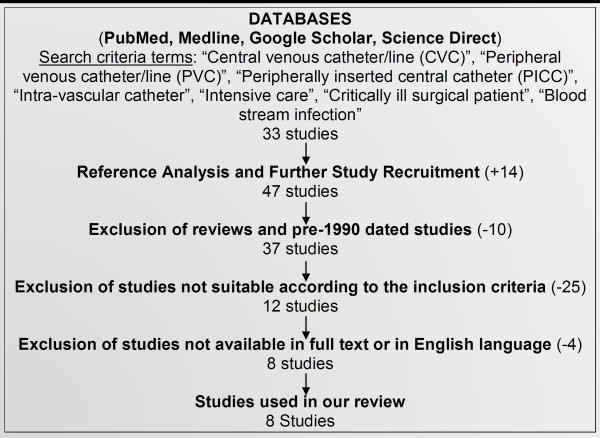
Flow chart illustrating search protocol and study selection.

An extensive systematic electronic search was carried out on the relevant databases including Pubmed, Pubmed Central, MEDLINE, Embase, Google Scholar and Science Direct. Due to the specificity of the review, various terms and Boolean operators were included in the search to ensure that relevant studies were not missed due to the search criteria (Figure 1). These terms included: “central venous catheter/line” (CVC), “peripheral venous catheter/line” (PVC), “peripherally inserted central catheter” (PICC), “surgical intensive care”, “critically ill surgical patient”, “blood stream infection”, “intra-vascular catheter”. This resulted in the retrieval of 47 studies. To supplement our search, we also reviewed the references of the above studies to identify additional articles that our search criteria may not have included. After our initial recruitment of studies, we excluded review papers, those that focused on medical patients and those dated pre-1990.

Articles were considered suitable for inclusion if they investigated catheter colonisation and catheter-related (CVC and/or PICC) bloodstream infection in critically ill surgical patients. Two independent reviewers were engaged in selecting appropriate articles in line with the above protocol.

The search protocol described above resulted in the selection of 8 original articles [7-14]; all exploring the occurrence of infections related to CVC and/or PICC in surgical patients. The various parameters evaluated as part of this review were as follows; study design, sample size, mean age & sex, APACHE Score, catheter indwelling time, antibiotic prophylaxis as well as definition and incidence of both catheter colonisation & CRBSI.

## Results

Our search protocol retrieved 8 articles published from 1999 to 2011 (see Table 1). Outcomes relating to CVC, including colonisation and CRBSI, were investigated in six studies; four of which were prospective cohort studies [8-11]^,^ one prospective longitudinal study [7] and one retrospective cohort study [12]. Outcomes relating only to PICCs were reported in one prospective randomised trial [14]. We found only one study [13], retrospective cohort in design, which compared CVC- and PICC-related complications in surgical intensive care units.

**Table 1:** Summary of study characteristics

**Study, Year**	**Study design**	**End point**	**Definition of Colonisation**	**Definition of CRBSI**
**Bijma**[7]**, 1999**	Prospective longitudinal cohort study	Impact of intervention plan on CVC colonisation and infection incidence	Growth of >15 cfu from the removed tip	Clinical signs of BSI in the absence of another focus of infection + both peripheral blood culture and catheter tip culture test positive for same organism
**Dimick**[8]**, 2001**	Prospective cohort study	Estimated increase in resource use associated with CRBSI of critically ill surgical patients after adjusting for severity of illness.	Growth of >15 cfu from the removed tip	Both peripheral blood culture and catheter tip culture test positive for same organism within 48 hours of each other
**Dimick**[9]**, 2003**	Prospective cohort study	Multipurpose CVC vs TPN CVC; risk factors, incidence and pathogens of CRBSI	Growth of >15 cfu from the removed tip	Both peripheral blood culture and catheter tip culture test positive for same organism within 48 hours of each other
**Sandoe**[10]**, 2003**	Prospective cohort study	Impact of extended routine perioperative antibiotic prophylaxis on incidence of CVC colonisation and infection	Growth of >15 cfu from the removed tip	Positive same-organism peripheral blood culture and catheter tip culture when catheter in situ
**Pawar**[11]**, 2004**	Prospective cohort study	Incidence, risk factors, outcome, and pathogens of CVC-BSI	Growth of <15 cfu from the removed tip	Clinical signs of BSI and both peripheral blood culture and catheter tip culture test positive for same organism OR resolution of fever after the removal of a CVC suspected of infection.
**Le Guillou**[12]**, 2011**	Retrospective cohort study	Proportion of surgical-site infections (SSIs) with possible attribution to CRBSI, risk factors associated with SSI after CRBSI.	Growth of >103 cfu/mL, and without clinical evidence of infection	BSI occurring 48 hours before/after catheter removal and positive culture with the same micro- organism of either (i) quantitative CVC culture >103 cfu/mL; (ii) positive culture from pus from insertion site; (iii) quantitative blood culture ratio CVC blood sample: peripheral blood sample >5; or (iv) differential time to positivity of blood cultures: CVC blood sample culture positive >2 hours before peripheral blood culture (blood samples drawn at the same time).
**Gunst**[13]**, 2011**	Retrospective cohort study	PICC VS CVC risk factors and incidence of CRBSI	Growth of >15 cfu from the removed tip	Both peripheral blood culture and catheter tip culture test positive for same organism

**Abbreviations:*CVC***: Central venous catheter; ***PICC***: Peripherally inserted central catheter; ***cfu***: Colony forming units; ***TPN***: Total parenteral nutrition; ***BSI***: Bloodstream infection.

Dimick et al. 2001 [8] and Dimick et al. 2003 [9] were both published using the same data set but looked at different outcomes. We treated them as one study in our pooled data and refer only to Dimick et al. 2003 [9], unless otherwise stated. Bijma et al [7]. compared simple infection control measures in a pre- and post-test group. Only post-test data was used in our analysis.

The population sample size and venous catheter number varied significantly between the studies we investigated from Le Guillou et al [12]. (n = 7557) to Miyagaki’s et al [14]. PICC study (n = 25). The only CVC and PICC comparative study, Gunst et al [13]., had a relatively small population size of 121 with 263 CVCs and 37 PICCs.

Our review was only concerned with venous catheter performance; we therefore excluded Sandoe’s et al. mixed colonisation data [10] and Dimick’s et al [9]. mixed CRBSI data. The former included 4 arterial catheters and we were unable to extract the CVC colonisation data whilst the latter included 348 pulmonary artery catheters and we were unable to extract CRBSI data. Furthermore, Pawar et al [11]., Miyagaki et al [14]. and Gunst et al [13]. failed to record colonisation incidence as part of their respective studies.

Prophylactic antibiotics were used in Sandoe et al [10]. and Pawar’s et al [11]. CVC study and Gunst’s et al [13]. CVC/PICC comparative study. In all instances prophylactic antibiotic treatment was reserved for CVC patients. Sandoe et al [10]. found no significant difference between short-course perioperative prophylaxis and extended prophylaxis in lowering the risk of catheter colonisation. These findings are in line with UK Department of Health guidelines [15], which do not recommend the use of antibiotics in preventing CRBSI during catheter placement.

Gunst et al [13]., our only comparative study, found that PICCs were associated with fewer CRBSI than CVCs in long-stay patients in surgical intensive care units (SICU). In the only randomised control trial Miyagaki et al [14]. compared the performance of two different PICC designs and reported a CRBSI incidence of 4%. However, a small sample group of 25 renders any conclusions open to question.

For CVC (Tables 2 and 3), catheter colonisation was reported to be as high as 12.88% [7] and as low as 8.4% [9] with a mean average for the pooled data of 8.93% [7,9,12]. Gunst et al [13]. and Pawar et al [11]. provided no data regarding colonisation incidence. The highest CRBSI incidence in the CVC studies was 4.9% [13], the lowest being 0% [10]. The mean average CVC CRBSI for the pooled data was 1.01% [7,10-13].

**Table 2:** Summary results of the selected studies

**Study, Year**	**Catheter Type Investigated**	**Population Size**	**Venous Catheter Number**	**Age (yr)**	**Sex (M/F)**	**APACHE II/III Score**	**Antibiotic Prophylaxis**	**Indwelling time: CRBSI group (Non-CRBSI group)**	**Colonisation (% total)**	**CRBSI (% total)**
**Bijma, 1999**[7]	CVC	128	n = 206	54	59/128	20 (II)	-	-	44 (21.36%)^b^	15 (7.28%)^b^
		140	n = 194	54	72/140	19 (II)	-	-	25 (12.88%)	8 (4.12%)
**Dimick, 2001 **[8]**/2003**[9]	PAC + CVC	260	n = 506	65	127/133	64 (III)	-	5 (3)	60 (8.4%)	17 (2.0%)^c^
**Sandoe, 2003**[10]	AC + CVC	179	n = 175	-	-	-	Yes	6 (4)	27 (15.1%)^d^	0 (0%)
**Pawar, 2004**[11]	CVC	1314	n = 1314^a^	58.4	1166/148	6.9 (II)	Yes	24.5 (6.1)	-	35 (2.7%)
**Guillou, 2011**[12]	CVC	7557	n = 7557^a^	65.1	5403/2154	-	-	-	653 (8.6%)	40 (0.5%)
**Gunst, 2011**[13]	CVC	121	n = 263	47	69/52	22 (II)	Yes	25 (16)	-	13 (4.9%)
	PICC		n = 37				No	19 (14)	-	1 (2.7%)
**Miyagaki, 2011**[14]	PICC	25	n = 25^a^	65.6	24/1	-	-	-	-	1 (4%)

**Abbreviations:*CVC***: Central venous catheter; ***PICC***: Peripherally inserted central catheter; ***AC***: Arterial catheter; ***PAC***: Pulmonary artery catheter; ***CRBSI*:** Catheter related bloodstream infection; ***APACHE***: Acute physiology and chronic health evaluation.

**Key:**^a^ Assumed patient number = catheter number ^b^ Bijma’s pre-intervention CVC colonisation and CRBSI data was excluded from pooled data. ^c^ Mixed PAC + CVC data so excluded from pooled data. ^d^ Mixed colonisation data (including 4 arterial catheters) excluded from pooled data.

**Table 3:** Pooled colonisation and CRBSI data

**Colonisation**				**CRBSI**			
	**Catheter No.**	**No. Colonisation**	**% Colonisation**		**Catheter No.**	**No. CRBSI**	**% CRBSI**
**CVC**	8257	738	8.94	**CVC**	9503	96	1.01
**PICC**	62	-	-	**PICC**	62	2	3.23

***CVC colonization*:** Pooled data includes Dimick and Guillou and Bijma’s post-intervention CVC colonisation data. Pawar and Gunst failed to record CVC colonisation incidence as part of their respective studies. ***PICC colonization*:** As Gunst and Miyagaki failed to record PICC colonisation incidence as part of their respective studies; we have no pooled PICC colonisation data. ***CVC CRBSI*:** We pooled the CVC-related bloodstream infection data from Bijma’s post-intervention group, Sandoe, Pawar, Guillou and Gunst’s studies. ***PICC CRBSI*:** We pooled Gunst and Miyagaki’s PICC-related bloodstream infection data. **Excluded data:** Bijma’s pre-intervention data was excluded from the pooled data as it established the baseline colonisation and infection incidence by which the efficacy of the intervention was measured in the post-intervention group. Dimick’s mixed catheter bloodstream infection data (that included 348 pulmonary artery catheters) was excluded from the pooled data, as we were unable to extract the CVC-related bloodstream infection incidence. Sandoe’s mixed colonisation data (that included 4 arterial catheters) was excluded from the pooled data, as we were unable to extract data relating to CVC colonisation incidence and catheter days.

The two sets of data pertaining to PICCs report a CRBSI incidence of 4% [13] and 2.7% [14] with a mean average for the pooled data of 3.23%. Neither, Gunst et al.13 nor Miyagaki et al [14]. provided any figures pertaining to colonisation incidence in PICCs. Thus our pooled data, contrary to Gunst’s et al [13]. findings, suggests that incidence of infection is lower in CVCs than PICCs.

## Discussion

Whilst it is expected that this systematic review’s search criteria located the most relevant papers, we cannot claim to have yielded a complete, thorough and comprehensive list. Only 8 papers met the inclusion and exclusion criteria and were deemed suitable for this review. Two studies [8,9] (deleted “of which”) used the same data set thus (deleted “therefore”) leaving us with 7 unique sets of data from which to draw our conclusions. One clear limitation and potential selection bias stems from our decision to only include studies published in the English literature.

A number of studies that investigated the comparative efficacy of PICC and CVC in critical patients had to be excluded on the grounds that data included medical patients. We excluded such studies to ensure that the conclusions drawn were accurate and a true representation of the surgical patient population.

There were some studies which investigated the use of CVCs and PICCs in surgical intensive care and seemed apparently suitable for our review but upon further analysis were excluded because although they referred to infections as a complication, they did not document catheter colonisation and/or CRBSI as one of their investigative parameters but rather focused on phlebitic, thrombotic and/or other such common complications.

Since the aim of the review was to compare CVC and PICC, a number of studies had to be excluded as they consisted of data relating to arterial catheters. We excluded these studies on the basis that arterial and venous catheters are entirely different entities with differing variable factors including haemodynamics and hence the sequelae of both are different. Studies which contained mixed venous and arterial catheter data but from which we could extract the venous catheter data were included, i.e. Sandoe et al [10]. and Dimick et al [9]. Further studies had to be excluded because although they referred to CVCs and PICCs, the findings were reported collectively and the two sets of data could not be demarcated from one another.

Only 1 of the 8 studies used a prospective randomised trial (PRT) study design; the others used a mixture of observational study designs. Whilst observational studies are prevalent in the infection control and critical care practice literature [16] they do limit, by design, the conclusions of our review. However it should also be noted that even PRTs are susceptible to bias, specifically those relating to the way the studies were conducted and data analysed [17].

All the authors followed published guidelines that clearly define positive catheter colonisation as either ≥15 colony forming units (CFU) by semi- quantitative culture [18] or ≥10 [3] CFU/mL by quantitative technique from culture of the distal end of the catheter [19]. The consistency in the definition of colonisation by the various studies reduced selection bias.

CRBSI is an ideal investigative parameter in comparing CVC and PICC performance as it represents the most serious form of venous catheter-related complication. However, the incidence of CRBSI is dependent upon the definition used. The Centre for Disease Control and Prevention (CDC) guidelines accepts various definitions for CRBSI [20,21]; these are further subcategorised into two broad groups, namely clinical definitions and surveillance definitions.

Clinical definitions of CRBSI include positive signs of bacteraemia with the catheter as the only focus of infection after meticulous exclusion of all other potential sources. In addition, both peripheral blood culture and catheter tip culture must test positive for the same organism.

The less stringent surveillance definitions of CRBSI include the resolution of fever after the removal of a CVC suspected of infection; such definitions greatly inflate the true incidence of CRBSI as the bacteraemia may be secondary to sources other than the catheter such as the postoperative surgical site, pancreatitis, urinary tract infection…etc.

Notwithstanding Miyagaki et al [14]. who, despite referring to CDC guidelines, failed to specify the CRBSI definition used, all the other studies used sub- definitions (Table 1) that fell within the scope of the more stringent category of ‘clinical definitions of CRBSI’. Dimick et al [9]., Sandoe et al [10]. and Gunst et al [13]. used the same definition of CRBSI, Bijma et al [7]. and Pawar et al [11]. used a different definition of CRBSI whilst Le Guillou et al [12]. used yet another. Also, Gunst’s et al [13]. retrospective design meant their study could not stringently follow the set definition.

It is important to be cognisant of the possibility that these varying definitions of CRBSI translate into differing thresholds for diagnosis of infection. (deleted “and” started a new sentence) Therefore the reported incidence of CRBSI would not only be different between studies but some may not have been a reflection of the true incidence of CRBSI. (deleted “and thus” started a new sentence). This could be pose another potential bias in our data analysis.

Our pooled data suggests that incidence of CRBSI is lower in CVCs (1.01%) than PICCs (3.23%) however Gunst et al [13]., our only comparative study, found the opposite to be true (CVC 4.9%: PICC 2.7%). It could be argued that the design of some of the CVC studies was such that they didn’t include risk factors for CRBSI and so under reported the true incidence of CRBSI thus conferring onto CVC an unwarranted level of safety with regards to BSI but without the missing data all inferences are inconclusive.

## APACHE score

There seems to be a positive correlation between the APACHE II score and CRBSI incidence in Bijma et al [7]., Pawar et al [11]. and Gunst et al [13]. These 3 studies reported CRBSI in line with the documented 1.3-14% [12,20] found in the literature based on mixed medical and surgical populations groups. Interestingly, both Sandoe et al [10]. and Le Guillou et al [12]. failed to capture the APACHE II score and reported a much lower CRBSI incidence of 0% and 0.53%, respectively. One explanation for the difference in CVC-RBSI between the two sets of CVC data (Bijma et al [7]., Pawar et al [11]., Gunst et al [13]. vs. Sandoe et al [10]. and Le Guillou et al [12].) could be that Sandoe’s et al [10]. and Le Guillou’s et al [12]. sample population included less severely ill surgical patients (APACHE II score). However this may not be the sole reason as Pawar et al [11]. identified the APACHE II score as only a univariant risk factor for CVC- RBSI.

## Catheter indwelling time

Dimick et al [9]. and Sandoe et al [10]. identified indwelling time to be an associated risk factor for CVC colonisation and infection whilst Gunst et al [13]. & Pawar et al [11]. found indwelling time to be a multivariate risk factor for CVC- RBSI. The detection of this strong risk association by Gunst et al [13]. and Pawar et al [11]. is due to the CVCs being left in place for at least 19 days; only Miyagaki et al [14]. had similar catheter days (median catheter dwell time = 16 days) although he failed to differentiate between infected and non-infected catheter days. The rest of the CVC studies had catheter days of 6 days or less and therefore cut short their studies before indwelling time could have an effect on CRBSI incidence.

In short, the reliable risk factors (whether univariate or multivariate) for CRBSI in surgical patients gleaned from our studies include indwelling time, use of TPN, the APACHE score and jugular vein insertion.

The apparent contradiction between Gunst’s et al [13]. comparative study that found CRBSI to be higher in CVCs than PICCs (4.9%: 2.7%) and our pooled data, which found the opposite (1.01%: 3.23%) could be explained by the cumulative effect of the following potential differences between Gunst et al [13]. and the other CVC studies; a less severely ill population sample (APACHE score) and shorter catheter days in CVC studies, varied definitions of colonisation & CRBSI, the accuracy of diagnostic methods deployed and, in the case of Sandoe et al [10]., the additional selection bias of eliminating patients that met the inclusion criteria (but were on another antibiotic therapy, thus potentially eliminating patients on suspected CRBSI treatment).

One caveat is that Gunst’s et al [13]. retrospective design meant his study couldn’t stringently follow the set definitions of colonisation and CRBSI and so there is the possibility that the true incidence of CRBSI was overestimated in his study.

Interestingly, Turcotte’s et al [3]. review of the literature found no significant difference in CRBSI incidence when comparing CVC and PICC, however his review included data from mixed medical and surgical populations and so does not directly relate to our review.

Probably the most interesting individual study is that of Dimick et al [8]. This study attempts to quantify the additional costs incurred between those with or without CRBSI. It suggests that a CRBSI significantly increases total health care costs from a median cost of $40313 without CRBSI vs. $102965 in those with CRBSI. Le Guillou et al [12]., has also suggested increased costs of up to 25% in those with surgical site infection in cardiac surgery (of which CRBSI is a significant risk factor). Dimick et al [8]. attributes the extra costs in patients with CRBSI to increased length of stay (room and board) as well as laboratory supply, and pharmacy costs. This study makes a good attempt to factor in analysis of the cost effectiveness of interventions and indeed concludes that given the increased costs incurred in patients with CRBSI, further preventative measures such as antiseptic-impregnated catheters can be justified on cost grounds alone. Dimick’s et al [8]. approach could be further developed by a paper exploring full cost-benefit analysis of all preventative methods for CRBSI in the surgical ICU setting to give a fuller picture of the costs of different CRBSI preventative measures and their relative effectiveness.

In Bijma et al [7]., a five-step prevention plan was found to significantly reduce the incidence of colonisation of CVCs however the observed reduction in CRBSI was not statistically significant (Table 4). The Dimick et al [9]. prevention approach of single lumen, single purpose (TPN) catheter placed in the subclavian vein and checked daily and maintained by a multidisciplinary team also significantly reduced the incidence of colonisation and resulted in no occurrence of CRBSI (Table 5). Both prevention approaches aim to prevent colonisation and CRBSI by eliminating known risk factors.

**Table 4:** Prevention plan as proposed by Bijma et al. 1999

	
1)	Introduction of hand disinfection with alcohol,
2)	Daily removal of a new nonwoven dressing,
3)	“One-bag” total parenteral nutrition (TPN) system,
4)	A new needless closed IV connection device, and
5)	Surveillance by an infection control practitioner

**Table 5:** Prevention plan as proposed by Dimick et al. 2003

	
1)	Catheters are inserted by 1 resident physician, who uses maximal barrier precautions,
2)	Single-lumen catheters only are used,
3)	The catheters are inserted in the subclavian site,
4)	The insertion sites are checked daily,
5)	TPN solution only is delivered through the catheter (to minimise hub manipulation), and
6)	Whenever a patient is transferred from another institution, blood samples are obtained through any indwelling catheters and are cultured; the cultures must be negative for pathogens before TPN therapy is started.

The only commonality between the two approaches is the daily maintenance and surveillance; 10 unique prevention pathways have been described and the individual efficacy of each yet to be elucidated. This has important implication for infection control policy particularly since non-evidence-based practices, such as extended prophylactic antibiotics treatment in lowering CRBSI, continue to be used [10] contrary to current UK guidelines [15]. Critical assessment of the efficacy of each described CRBSI preventive intervention in surgical patients using randomised controlled trials remain essential in order to drawing up the most cost-effective evidence-based CRBSI prevention plan.

**Figure 2:**
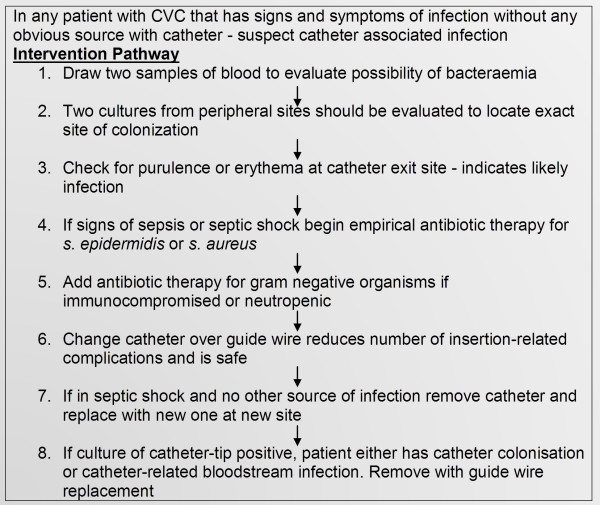
Pathway to Intervention (adapted from McGee and Gould 2003).

Mixed medical/surgical studies in an ICU setting have found the duration of catheterisation to be associated with an increased incidence of CRBSI. The risk of CRBSI is low until the fifth to seventh days of catheterisation, after which there is almost a fourfold increase in infection rates between days 7 to 14 and a fivefold increase thereafter [22]. Therefore, venous catheters ought to be removed as soon as they are no longer clinically needed, since the probability of CRBSI increases over time. Gunst’s et al [13]. non-randomised study went on to suggest the substitution of PICC for CVC in long-stay SICU patients may further reduce incidence of CRBSI but admitted such recommendations need validation via large prospective studies. McGee and Gould review [23] suggested pathway for intervention (Figure 2).

## Conclusion

Although our systematic review search protocol may not have yielded an exhaustive list we have identified a key deficiency in the literature namely a paucity of studies investigating the incidence of CVC- and PICC-related bloodstream infection in exclusively critically ill surgical populations.

In summary, the diverse definitions for the diagnosis of CRBSI along with the vastly different sample size and extremely small PICC population size has, predictably, yielded inconsistent findings. Our current understanding is still limited; the studies we have identified do point us towards some tentative understanding of CVC/PICC performance with regards to CRBSI but much still remains inconclusive.

Critically ill surgical patients not only comprise an important subset of those in the ICU but they also experience prolonged stays and often require TPN catheter intervention. Given that CRBSI accounts for up to 20% of hospital- acquired infections in the UK and is associated with both increased ICU stay and mortality, there is an imperative need for large scale randomised prospective studies investigating the incidence of CVC- and PICC-related infections in critically ill surgical patients in order to elucidate evidence-based guidelines for the prevention and intervention of CRBSI.

Furthermore, future studies should adhere to the same protocol with respect to study design, catheter indwelling time and CRBSI definition so that meaningful and valid comparison and appraisals of research literature can take place.

## Competing interests

The authors declare that they have no competing interests.

## Authors’ contribution

All authors contributed to conception and design, carried out the literature research, manuscript preparation and manuscript review. All authors read and approved the final manuscript.
